# For Diphtheria

**Published:** 1874-01

**Authors:** L. B. Bouchelle

**Affiliations:** Midville, Burke County, Georgia


For Diphtheria.—This terrible scourge to our race has been the subject of much theorizing and experimentation, and has been as incorrigible as any disease with which the profession has to contend. In earlier years my treatment was an emetic, a dose of calomel (cathartic dose), quinine, and local astringents to fauces, and in obstinate cases, blisters; and the treatment was very satisfactory if the case was seen as early as twenty-four hours after its onset. But of late I have had occasion to change to the following, with unqualified satisfaction, viz.: Water, Oss.; ipecac, grs. xx.; quinine, grs. xx.; carbolic acid, gtts. xxx. to xl. M. After mingling well, give a tablespoonful every half hour till the patient vomits freely; then give a teaspoonful every one to two hours for a day or two. The dose named will do for persons from ten years up; under ten years, less will do. This will be found—has proved so in my hands—an excellent prescription in scarlatina and other ulcerations about the throat.

L. B. Bouchelle, M.D.

Midville, Burke County, Georgia.

